# Canine Papillomavirus 2 E6 Does Not Interfere With UVB-Induced Upregulation of p53 and p53-Regulated Genes

**DOI:** 10.3389/fvets.2021.570982

**Published:** 2021-03-03

**Authors:** Sarah Quinlan, Susan May, Ryan Weeks, Hang Yuan, Jennifer Luff

**Affiliations:** ^1^Department of Population Health and Pathobiology, College of Veterinary Medicine, North Carolina State University, Raleigh, NC, United States; ^2^Department of Pathology, Georgetown University Medical Center, Washington, DC, United States

**Keywords:** canine papillomavirus type 2, E6, p53, LncRNA-p21, bak, bax

## Abstract

Cutaneous papillomaviruses are oncogenic viruses that cause severe, persistent infections that can develop into skin cancers within ultraviolet (UV)-exposed skin of immunodeficient individuals, such as those with X-linked severe combined immunodeficiency (XSCID). A canine research model of XSCID exhibits a similar phenotype; these dogs develop severe canine papillomavirus 2 (CPV2) infections that often progress to cancer. Thus, the dog is a natural, spontaneous model to investigate cutaneous papillomavirus infections in immunodeficient patients. The human papillomavirus oncogene E6 contributes to cancer development, in part, by initiating degradation of the tumor suppressor protein p53, or by inhibiting upregulation of p53-dependent genes required within the cell growth arrest and apoptotic pathways, thereby leading to an accumulation of DNA damage required for oncogenesis. Currently, little is known about CPV2, and how it promotes cancer development. The aim of this study was to determine if CPV2 oncogene E6 similarly affects p53 upon activation by UV radiation, as well as the downstream p53-regulated genes necessary to control growth arrest and apoptosis. We determined that cutaneous CPV2 E6 does not degrade p53, or interfere with the upregulation of p53-regulated genes p21, Bax, Bak, or lncRNA-p21, suggesting that CPV2 may use a p53-independent mechanism to contribute to oncogenesis.

## Introduction

Papillomaviruses (PV) are host specific, epitheliotropic, circular double-stranded DNA viruses that are widely prevalent among mammals. There are over 200 human papillomavirus (HPV) types, which infect mucosal or cutaneous epithelium (1). Certain high risk (HR) mucosal HPVs are causally associated with anogenital and oropharyngeal cancers. Other HPV types infect skin, and normally cause asymptomatic infections or benign lesions or warts. However, in immunocompromised individuals, such as those on immunosuppressive therapies due to organ transplantation, those infected with human immunodeficiency virus, or those with genetic immunodeficiencies such as epidermodysplasia verruciformis or X-linked severe combined immunodeficiency (XSCID), some of these cutaneous HPVs have been implicated in the development of severe, persistent cutaneous PV infections that, along with UV radiation, can lead to non-melanoma skin cancers on sun-exposed skin sites (1–5). In long- term follow-up studies on XSCID patients, for example, approximately 50% of these patients develop severe cutaneous PV infections at sun exposed sites that are refractory to treatment and can progress to cancer (6–8).

A similar increased risk of severe cutaneous PV infections has been observed in a research colony of dogs with XSCID (9). These dogs are used as a critical animal model for human XSCID and exhibit a similar clinical and immunological phenotype as their human XSCID counterparts (9–12). Approximately 70% of these dogs develop severe cutaneous PV infections that can progress to metastatic cancer in ~70% of affected dogs (9). Canine papillomavirus 2 (CPV2) is the virus associated with these cutaneous infections in XSCID dogs (9, 13). Because canine and human PVs share key biological characteristics and mechanisms of action, they are an ideal, natural model to study cutaneous PV virus-host interactions (9–11, 13, 14).

The genomes of both mucosal and cutaneous PV types code for early genes which are involved in viral replication and transcription (15). One of the early genes, E6, from mucosal HR HPV promotes degradation and inactivation of the host tumor suppressor gene p53, which has a prominent role in regulating cellular apoptosis, growth arrest, and DNA repair (15, 16). Upon DNA damage by UVB radiation for example, p53 is upregulated, and will either induce cell cycle arrest to allow DNA repair, or if the damage is too extensive, initiate apoptotic pathways. High risk HPV E6 recruits p53 to E6-associated-protein (E6AP) for ubiquitin-mediated proteosomal degradation (17). The lack of p53 then leads to a reduction in downstream p53-regulated genes such as p21 and Bak, thereby preventing growth inhibition and apoptosis, leading to the accumulation of DNA damage, and cancer-inducing genetic instability. Cutaneous HPV E6 also plays a part in cancer development by exacerbating the accumulation of UV radiation-induced DNA damage, albeit through differing mechanisms. E6 from different cutaneous HPV types does not initiate degradation of p53, but some can still interfere with growth arrest and apoptosis (18). For example, E6 associates with and promotes the degradation of p300, a histone acetyltransferase, which then prevents p53 acetylation, causing destabilization and inactivation (19). E6 from some cutaneous HPVs also targets the proapoptotic protein Bak for degradation, preventing UV-induced apoptosis, and allowing survival of DNA damaged cells, which can progress to non-melanoma skin cancer (18, 20, 21).

These cell cycle and apoptotic pathways commonly rely on p53-induced genes to translate effector proteins to carry out these functions. However, there are RNA transcripts that lack protein coding capabilities, but still have an effect on cellular function through transcriptional regulation, RNA processing, modification or translation (22). Many of these non-coding RNA are long, >200 nucleotides, and are called long, non-coding RNAs (lncRNA). LncRNA-p21 is a non-coding RNA located ~15 kb upstream from the *Cdkn1a* (p21) gene. It has been shown that lncRNA-p21 is highly inducible by UVB in keratinocytes, and is a p53 responsive gene that regulates apoptosis in UVB-treated mouse and human keratinocytes (22). Canine lncRNA-p21 has not been previously characterized.

As described above, the effect of mucosal and cutaneous HPV E6 on p53 and its effector functions are well established. We have previously shown that CPV2 E6 impacts expression of type I and III interferons and interferon stimulated genes (23). However, nothing is currently known about CPV2 E6 and its effect on cell cycle regulation or apoptosis, so the objective of this study was to determine how CPV2 E6's behavior in this manner compares to the human E6 oncogene. To this aim, we examined the response of p53 and p53-regulated genes p21, Bak, Bax, and lncRNA-p21 within UV-exposed normal canine keratinocytes, and with keratinocytes that expressed CPV2 E6. We determined that cutaneous CPV2 E6 does not degrade p53, nor interfere with the expression of p21, Bax, Bak, or lncRNA-p21. As CPV2 is associated with metastatic cutaneous carcinomas in these immunodeficient dogs, it is likely there is another as yet undetermined mechanism not dependent on degradation of p53 or Bak that leads to oncogenesis.

## Materials and Methods

### Plasmids and Retrovirus Transduction

Wild-type CPV2 E6 gene was amplified from the viral genome by PCR and subcloned into the retrovirus vector pLSXN at the sites EcoRI and BamH I (Takara Bio USA Inc., MountainView, CA) as previously described (14, 23). Retrovirus stocks were prepared by transfecting the retrovirus packaging cell line SD3443 cells with vector only or CPV2 E6 retrovirus constructs using Fugene (Roche applied science, Penzberg, Germany) as specified by the manufacturer and as previously described (14, 23). Culture supernatants containing retrovirus were collected after 48 h post-transfection and viral titers determined using 3T3 cells. Canine primary epidermal keratinocytes (CPEKs) (ZenBio, Research Triangle Park, NC) (24), which were isolated from normal tissue and have not been actively transformed, were infected at a multiplicity of 10 PFU/cell with retrovirus containing vector only or wild type E6. Retrovirus-infected cells were selected in G418 (50 ng/ml) for 2 days. Amplicons from CPV2 E6 expressing CPEKs were purified and sequenced to ensure integrity of transduced constructs.

### Identification of Canine lncRNA-p21

Human lncRNA-p21 is 3,898 bp in length and located ~15 kb upstream from the Cdkn1a (p21) gene [GenBank accession number: KU881768.1]. A 1,048 bp portion of canine lncRNA-p21 was identified by aligning the human sequence to the canine genome (CanFam 3.1) using the NCBI nucleotide BLAST tool. This canine lncRNA-p21 sequence is 73% identical to human lncRNA-p21, and covers between 164 and 1,176 of the human sequence. This canine sequence is located on chromosome 12 [573,7376–573,6329], ~15 kb upstream from the Cdkn1a (p21) gene. The nucleotide sequence was submitted to Genbank (accession number: MT333742).

### Cell Culture

CPEKs were maintained in canine keratinocyte media CNT-09 (ZenBio) with the addition of penicillin-streptomycin antibiotics (Sigma-Aldrich, St. Louis, MO). For experiments, CPEKs were seeded into 6 well plates or 100 mm dishes, incubated at 37°C and 5% CO_2_ overnight, before exposure to UVB. For cells in culture, the medium was removed, and the cells washed in PBS before exposure to UVB for 18 s at a distance of 15.3 cm (10 mjoules/cm^2^) from the UV source (Spectronics Corporation, Westbury, NY, EB-280-C; 8 W, 312 nm). This lamp has a filter that blocks out all other wavelengths. The light intensity of the lamp was measured by the IL-1700 Research Radiometer (International Light Technology, Peabody, MA) equipped with an SED 240 sensor. After irradiation, fresh medium was added to the wells before the cells were lysed for protein or RNA extraction at appropriate time points.

### Reverse Transcription PCR

Total RNA was extracted from cell lysates using a commercially available kit (RNeasy mini kit, Qiagen, Hilden, Germany). Complementary DNA (cDNA) was generated (QuanitiTect, Qiagen) from 1,000 ng of total RNA following manufacturer's recommended protocol. The resulting 20 μl cDNA was diluted with 180 μl RNase free water. Real time PCR using SYBR green detection (Qiagen) was performed for the following genes: Ribosomal Protein L13a (RPL13A), CPV2 E6, and lncRNA-p21. Primer pairs for RPL13A were designed based upon the GenBank sequence (accession number: NM_001313766.1) using Primer 3 software. Primer pairs for CPV2 E6 were designed based upon the GenBank sequence (accession number: NC_006564.1) using Primer 3 software. Primer pairs for lncRNA-p21 were designed based upon the nucleotide sequence submitted to GenBank (accession number: MT333742). Primer efficiencies were determined using a standard curve generated using purified PCR product. Primer sequences and efficiencies are listed in Table 1. Reaction mixtures contained 12.5 μl QuantiTech SYBR green master mix (Qiagen), 0.2 μM concentration of the forward and reverse primers, 10 μl of diluted cDNA (1:10), and enough RNase free water to yield a total of 25 μl reaction mixture. All real time PCR reactions were performed in 96-well plates on the Roche 480 Light Cycler system. The expression of individual genes was normalized to expression of the reference gene and graphed as the relative fold change between non-UVB exposed and UVB exposed samples based upon the 2-ΔΔCq method.

**Table 1:** Primer sets and primer efficiency for RT-qPCR.

**Target gene**	**Primer sequence forward and reverse (5'-−3')**	**Efficiency (%)**
*RPL13A*	TGGGCCGGAAGGTTGTAGTCGT	99
	TTGCGGAGGAAGGCCAGGTAATTCA	
*lncRNA-p21*	GCAAGTCTCTCCCCAAATCG	94
	CGGTTATGGATGCCGTCAAG	
*CPV2 E6*	ATATTTATGAAACCGTTAGCC	99
	CGCAGCTGTCACAAGTGTTCC	

*RPL13A (ribosomal protein L13a)*.

### Preparation of Whole Cell Lysates

Whole-cell lysates were prepared by washing cells in cold PBS, harvested by scraping, and collected by centrifugation. RIPA buffer (Sigma-Aldrich) containing protease and phosphatase inhibitors (Thermo Fisher Scientific, Waltham, MA) was added to the cell pellet, incubated for 5 min on ice, before centrifugation at 14,000 × g for 10 min. Supernatants were stored at −80°C until use. Protein concentration was determine using a bovine serum albumin protein assay kit (Thermo Fisher Scientific).

### Cell Counting Kit-8 (CCK8) Assay

The cell viability after UVB exposure was assessed in normal CPEKs and CPEKs expressing CPV2 E6. Approximately 1.5 × 10^5^ cells were seeded in triplicate into multiple wells of 6-well tissue culture plates and incubated at 37°C and 5% CO_2_ for 24 h. After 24 h, the cells were exposed to UVB as described above. At the appropriate time post-irradiation, culture media was removed from the wells, replaced with 750 μl of fresh CNT-09, and 10 μl of CCK8 reagent (Dojindo Molecular Technologies, Rockville, MD) was added to the wells. The plates were incubated at 37°C and 5% CO_2_ before 100 μl of culture media was assayed in triplicate in a 96-well plate. The optical density (OD) value in each well was detected at a wavelength of 450 nm at indicated time points post-irradiation using a microplate reader (Sunrise, Tecan, Switzerland) and data analysis software (Magellan, Tecan).

### Western Blot Analysis

Equal amounts of protein were dissolved in 2X Laemmli sample buffer (BioRad Laboratories, Hercules, CA), boiled, and separated by sodium dodecyl sulfate (SDS)-polyacrylamide gel electrophoresis. The separated proteins were transferred to a nitrocellulose membrane (Biorad). Following incubation for 1 h in 5% milk blocking buffer, the membranes were probed with rabbit polyclonal antibody against p21 (Cat #: 10355-1, Proteintech, Rosemont, IL) at 1:2,000 dilution; rabbit polyclonal antibody against Bax (Cat #: ab104156, Abcam, Cambridge, MA) at 1:1,000 dilution; rabbit polyclonal antibody against Bak (Cat #: ab69404, Abcam) at 1:1,000 dilution; and mouse monoclonal antibody against p53 (Cat #: ab26, Abcam) at 1:1,000 dilution overnight at 4°C. The membranes were washed and then probed with a horseradish peroxidase-conjugated rabbit (Life Technology, Carlsbad, CA) or mouse (Pierce Biotechnology Inc., Thermo Fisher Scientific) secondary antibody at 1:5,000 dilution for 1 h. Detection was made using a chemiluminescence reagent (GE Healthcare, Chicago, IL), followed by exposure of the membrane to film. Membranes were probed with an antibody to β-actin directly conjugated to horseradish peroxidase (Abcam) to confirm equal protein loading.

### Statistical Analysis

Statistical analysis and graphical presentation were performed using Graph Pad Prism 7 software (GraphPad software, San Diego, CA). Normal distribution was determined using D'Agostino & Pearson test. Mean values for relative gene expression were compared using one-way ANOVA, followed by Dunnett's multiple comparisons test where appropriate. A *p* < 0.05 was considered significant.

## Results and Discussion

### p53 and p53-Regulated Genes Are Upregulated in Normal Canine Keratinocytes After UVB Exposure

**Figure 1:**
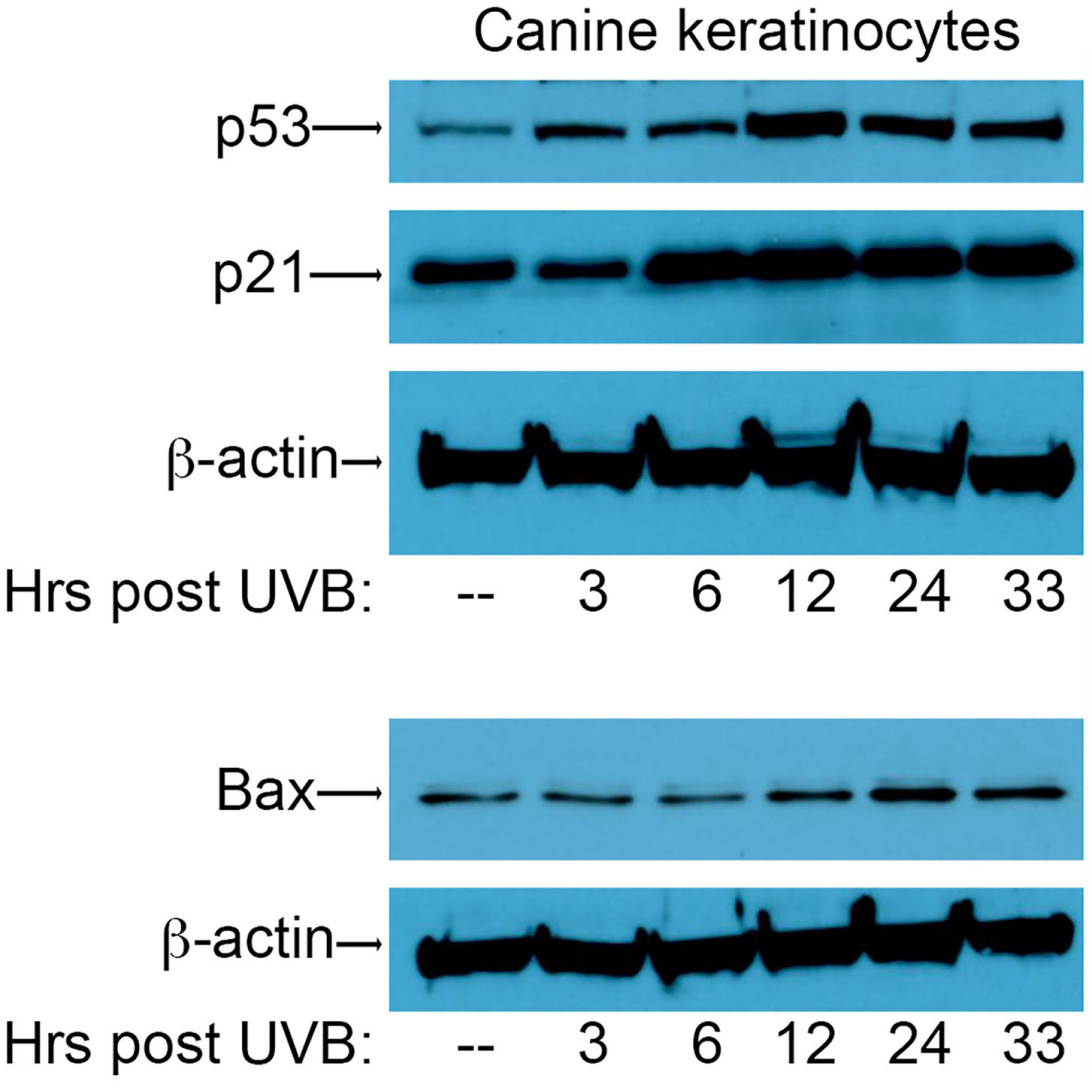
Upregulation of p53 and p53-regulated genes *p21* and *Bax* in canine keratinocytes in response to exposure to ultraviolet B (UVB) light. Normal canine keratinocytes were exposed to UVB (10 mJ/cm^2^) and cell lysates collected at different times (hours) post-irradiation. Proteins were separated by SDS-PAGE and analyzed by immunoblotting with antibodies directed against p53, p21, and Bax. β-actin was used a loading control. One representative experiment of three is shown.

In order to assess the effect of CPV2 E6 on activation of the p53 pathway, we first needed to establish conditions for the induction of DNA damage using UVB in canine keratinocytes to assess p53 responses. To this end, primary canine keratinocytes (CPEKs) were exposed to a single dose of UVB (10 mJ/cm^2^) before protein isolation at 3, 6, 12, 24, and 33 h post-UVB treatment. Equal amounts of protein extract were fractionated by SDS-PAGE and analyzed by western blotting using antibodies specific for p53, p21, and Bax. Expression of p53 was starting to increase 3 h post-irradiation, peaked at 12 h post-irradiation, and remained elevated at 33 h post-irradiation (Figure 1). Expression of p21 was increased by 6 h post-irradiation, and remained elevated at 33 h post-irradiation (Figure 1). Expression of Bax increased at 12 h post-irradiation, peaked at 24 h post-irradiation, and remained slightly elevated at 33 h post-irradiation (Figure 1). Expression of β-actin confirmed equal protein loading between the samples (Figure 1). Given these results, we chose 12 h and 24 h post-UVB exposure as optimal time points to assess upregulation of p53 and p53-regulated genes in CPV2 E6 expressing CPEKs.

### lncRNA-p21 Is Upregulated in Normal Canine Keratinocytes After UVB Exposure

**Figure 2:**
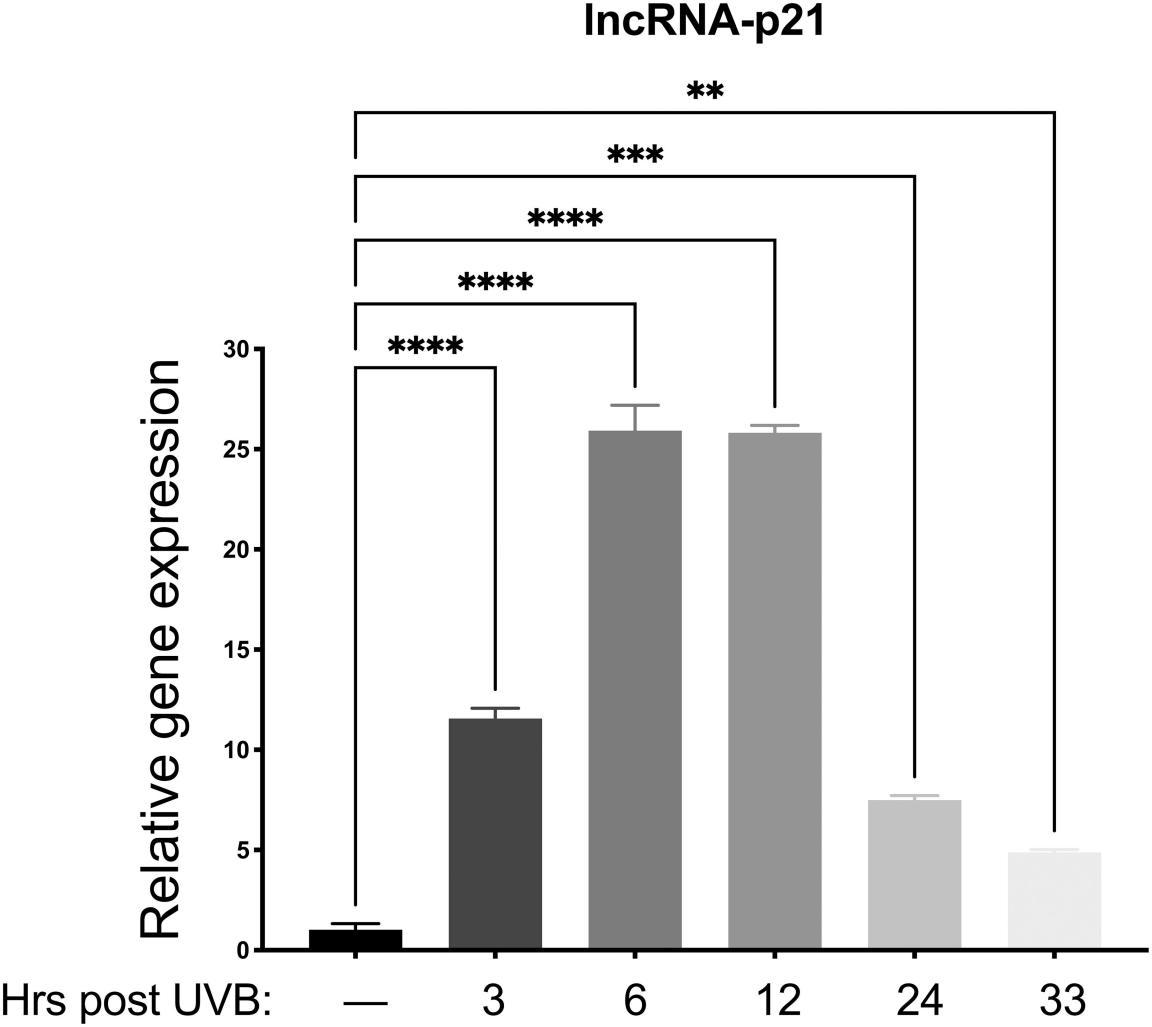
Upregulation of long non-coding (lnc.) RNA-p21 in canine keratinocytes in response to exposure to ultraviolet B (UVB) light. Normal canine keratinocytes were exposed to UVB (10 mJ/cm^2^) and cell lysates collected at different times points post-irradiation for RNA isolation. Real time PCR was performed to determine expression of lncRNA-p21. Resulting Cq values were normalized to the Cq value of the reference gene and calibrated to transcript levels in non-UVB exposed keratinocytes. Experiments were performed in duplicate and repeated in three independent experiments. Results of one representative experiment is shown and expressed as mean ± SD. **p < 0.01; ***p < 0.001; ****p < 0.0001.

Preliminary data from our lab revealed only minimal mRNA upregulation of known p53 regulated genes (p21, MDM2, Bak, and Bax) in vector only control cells. Therefore, we chose to examine *lncRNA-p21*, as this gene is a known p53-induced gene responsive to UVB in mice and human keratinocytes (22), and preliminary data showed upregulation in canine keratinocytes after UVB exposure. Thus, we aimed to determine which time points would be best to analyze gene expression after UVB exposure. To this aim, normal primary canine keratinocytes were exposed to a single dose of UVB (10 mJ/cm^2^) before RNA isolation at 3, 6, 12, 24, and 33 h post-UVB treatment. We determined that expression of lncRNA-p21 was increasing by 3 h post-irradiation, peaked around 6–12 h post-irradiation, and was still slightly elevated at 33 h post-irradiation (Figure 2). Given these results, we chose 12 h and 24 h post-UVB exposure as optimal time points to assess upregulation of lncRNA-p21. Thus, similar to human and mouse, canine lncRNA-p21 appears to be responsive to UVB in canine keratinocytes. In human and mouse keratinocytes, this UVB-induced lncRNA-p21 upregulation is dependent upon p53 (22); and, given the similarity between human and canine lncRNA-p21, and its response to UVB, it is likely, albeit not substantiated, that canine lncRNA-p21 is also regulated by p53.

### p53 and p53-Regulated Genes Are Upregulated in Canine Keratinocytes Expressing CPV2 E6 After UVB Exposure

E6 genes from some high-risk mucosal papillomaviruses degrade p53 and prevent upregulation of p53 regulated genes (15, 25). E6 from cutaneous papillomaviruses do not typically degrade p53, but some cutaneous papillomavirus E6 genes can prevent upregulation of some p53 regulated genes (26) or target the proapoptotic gene Bak for degradation (20, 21). To investigate if CPV2 E6 can disrupt p53 signaling, canine keratinocytes were generated that express the E6 gene from CPV2 using retrovirus transduction. CPV2 E6 expression was confirmed using real time reverse transcriptase PCR (RT-qPCR), as antibodies to detect these proteins are not available. Cq values for CPV2 E6 averaged 16.6 (reference gene Cq values averaging 14.7) in CPV2 E6 expressing keratinocytes.

**Figure 3:**
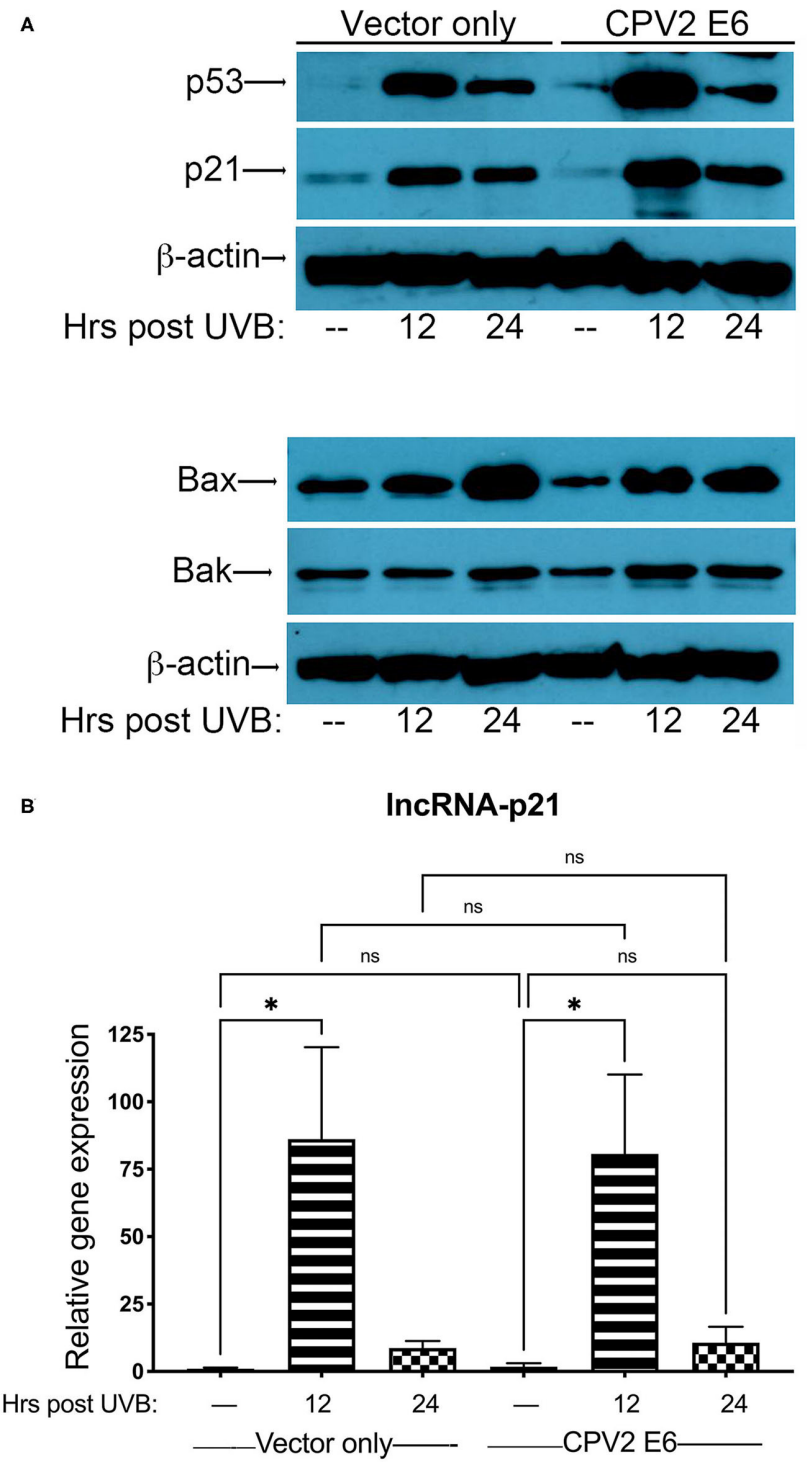
Upregulation of p53 and p53-regulated genes in UVB-exposed canine keratinocytes expressing CPV2 E6. Canine keratinocytes expressing either vector only or CPV2 E6 were exposed to UVB (10 mJ/cm^2^) and cell lysates collected at 12 and 24 h post-irradiation. **(A)** Proteins were separated by SDS-PAGE and analyzed by immunoblotting with antibodies directed against p53, p21, Bak, and Bax. β-actin was used a loading control. One representative experiment of three is shown. **(B)** Real time PCR was performed to determine expression of lncRNA-p21. Resulting Cq values were normalized to the Cq value of the reference gene and calibrated to transcript levels in vector only non-UVB exposed cells. Experiments were performed in duplicate and repeated in three independent experiments. One representative experiment of three is shown. Results are expressed as mean ± SD. *p < 0.05.

Next, we exposed either vector only or CPV2 E6-expressing canine keratinocytes to a single dose of UVB (10 mJ/cm^2^) before protein and RNA isolation at 12 and 24 h post-UVB treatment. Equal amounts of protein extract were fractionated by SDS-PAGE and analyzed by western blotting using antibodies specific for p53, p21, Bak, and Bax. Expression of p53 was upregulated in both vector only and CPV2 E6 expressing cells at 12 and 24 h post-irradiation (Figure 3A). p21 was also upregulated in both vector only and CPV2 E6 expressing keratinocytes at 12 and 24 h post-irradiation; Bax was upregulated in both vector only and CPV2 E6 expressing keratinocytes at 24 h post-irradiation (Figure 3A). Expression of Bak was slightly elevated in vector only cells at 24 h and in CPV2 E6 expressing cells at 12 and 24 h post-UVB exposure. As protein expression of CPV2 E6 could not be verified due to lack of antibodies, we confirmed these results for p53 and Bak using CPEKs that express a 3xFLAG epitope on the N-terminus of CPV2 E6 (Supplementary File 1).

Expression of lncRNA-p21 was highest at 12 h post-irradiation in both vector only and CPV2 E6 expressing keratinocytes (Figure 3B). Thus, we found there was no degradation of p53 or inhibition of upregulation of p21, Bax, Bak, or lncRNA-p21 by CPV2 E6. Similar to other cutaneous papillomaviruses (18, 21, 26), we found that p53 is not degraded in CPV2 E6 expressing cells. We found that CPV2 E6 does not block induction of p21, similar to some other cutaneous papillomaviruses where E6 does not prevent induction of p21 (26). Bak was not degraded before or after UVB exposure, unlike E6 from cutaneous HPVs that promote proteolytic degradation of Bak and evasion of UVB-induced apoptosis (20, 21). We had examined mRNA induction of other known p53 regulated genes (p21, MDM2, Bak, and Bax), but upregulation was minimal in vector only control cells; thus, we chose to examine lncRNA-p21, as this gene was significantly upregulated after UVB exposure and is a known p53 regulated gene in humans and mice.

### Cell Proliferation Is Inhibited After UVB Exposure in Canine Keratinocytes Expressing CPV2 E6

**Figure 4:**
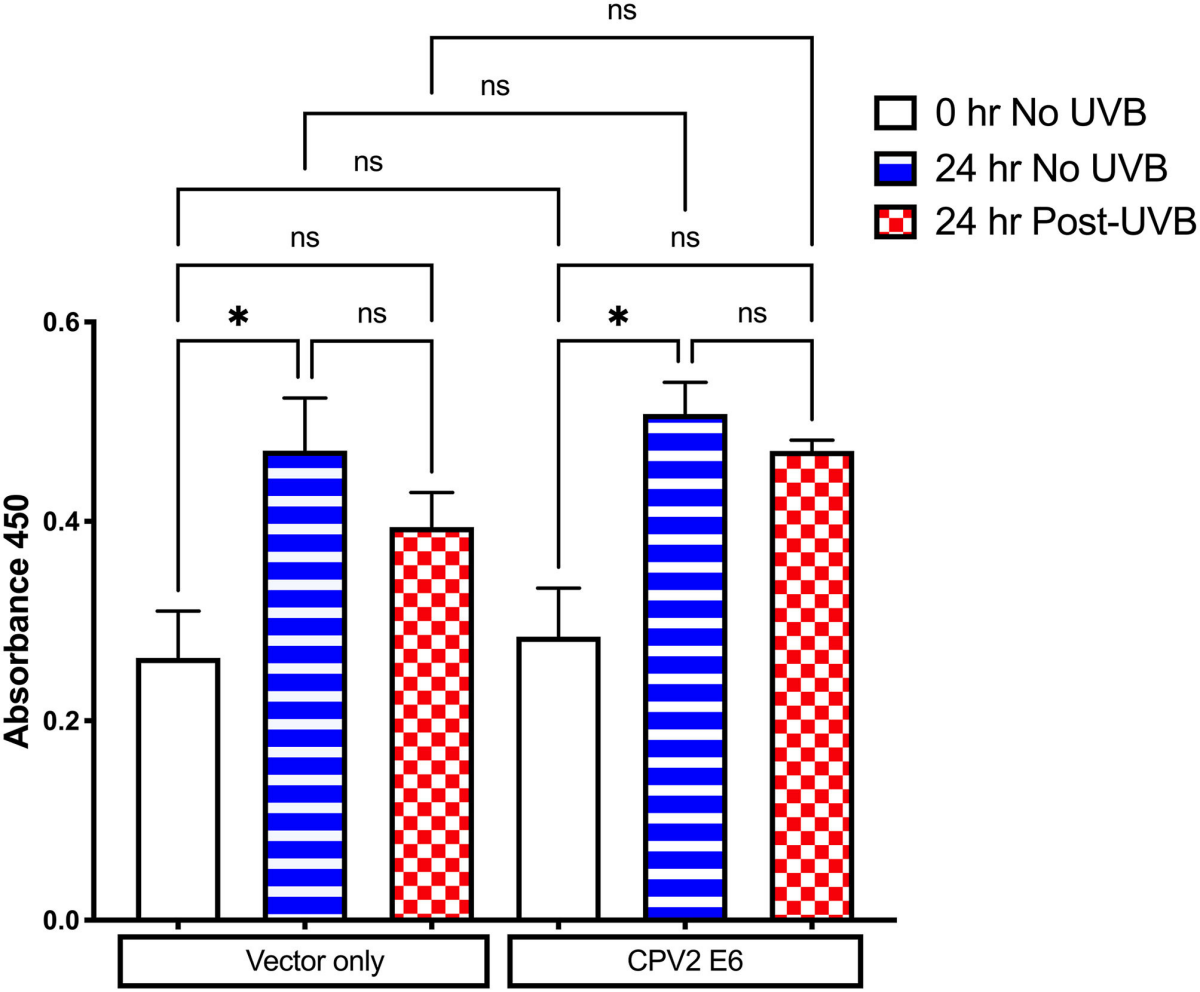
CCK8 proliferation assay in UVB-exposed canine keratinocytes expressing CPV2 E6. Canine keratinocytes expressing either vector only or CPV2 E6 were seeded in 6 well tissue culture plates, cultured overnight, and then exposed to UVB (10 mJ/cm^2^). CCK8 assay was performed at time 0, and then 24 h post-irradiation. Experiment was performed in triplicate and repeated three independent times. Results are expressed as mean ± SEM. *p < 0.05.

E6 from some cutaneous papillomaviruses can attenuate cell death and proliferation arrest after UVB exposure. The CCK-8 assay was used to evaluate cell viability and proliferation following UVB exposure in CPEKs expressing vector only or CPV2 E6. As shown in Figure 4, there was cellular proliferation at 24 h (increased OD value) in non-UVB exposed CPEKs expressing both vector only and CPV2 E6, demonstrating that CPV2 E6 expressing cells do not seem to have a growth advantage at baseline. After UVB exposure, proliferation of both vector only and CPV2 E6 expressing cells was decreased, although this was not statistically significant, compared to non-UVB exposed cells; while CPV2 E6 expressing cells had a trend toward slightly higher proliferation compared to vector only expressing cells, this did not reach significance. Overall, these data suggest that CPV2 E6 does not confer a significant growth advantage to keratinocytes.

Like high-risk HPVs, the E6 protein of CPV2 contains a carboxyl terminal PDZ domain protein binding motif (D-S-C-V-COOH), suggesting that CPV2 E6 may behave like high-risk HPVs which can bind to PDZ proteins and, in some cases, lead to their E6AP-mediated ubiquitination and proteolysis (13). We found, however, that CPV2 E6 does not degrade p53 like the high-risk HPVs types, nor does it interfere with UVB-induced upregulation of p21, Bak, or Bax. Cutaneous beta-HPV types are associated with a rare genodermatosis epidermodysplasia verruciformis that results in early development of disseminated flat warts that can progress to cancer at UVB exposed sites (27). These beta-HPV types are unable to degrade p53 but can degrade Bak or prevent its accumulation after UVB exposure to evade apoptosis (20, 21). CPV2 E6, unlike these beta-HPV types, does not degrade Bak or prevent its accumulation after UVB exposure.

These data collectively indicate that while some papillomaviruses can decrease expression of p53, some p53 regulated genes, and the proapoptotic gene Bak, this does not seem to be true for CPV2 E6. Given these results, it is likely there is another, as yet undetermined, mechanism not dependent on degradation of p53 or Bak leading to oncogenesis in these immunodeficient dogs. In the general dog population, CPV2 induces cutaneous papillomas within older dogs which most often regress spontaneously and is not associated with progression to cancer (28). Thus, it is plausible that CPV2 is a low-risk papillomavirus type in the general dog population, but causes metastatic cancers in dogs with a specific immunodeficiency. Thus far, this has been limited to dogs with the immunodeficiency XSCID (9). A similar predisposition to cutaneous papillomavirus infections is seen in long-term follow-up studies on humans with XSCID (6, 7, 29), and it is likely that host-factors, in addition to viral factors, play a significant role in cancer development in these patients.

## Data Availability Statement

The nucleotide sequence generated for this study can be found in NCBI Genbank, NCBI Accession No. MT333742. All other raw data supporting the conclusions of this article will be made available by the authors, without undue reservation.

## Author Contributions

JL designed, conducted the study, and edited the manuscript. SQ analyzed data and drafted the manuscript. RW and SM conducted the study. HY designed and conducted the study. All authors reviewed and approved the final manuscript.

## Conflict of Interest

The authors declare that the research was conducted in the absence of any commercial or financial relationships that could be construed as a potential conflict of interest.
