# Supplementary material for: Canine Papillomavirus 2 E6 Does Not Interfere With UVB-Induced Upregulation of p53 and p53-Regulated Genes
Source: Front Vet Sci. 2021 Mar 3;8:570982. doi: 10.3389/fvets.2021.570982 (PMC7965962; doi:10.3389/fvets.2021.570982)
Supplement: Supplementary file 1 [file Data_Sheet_1.pdf]

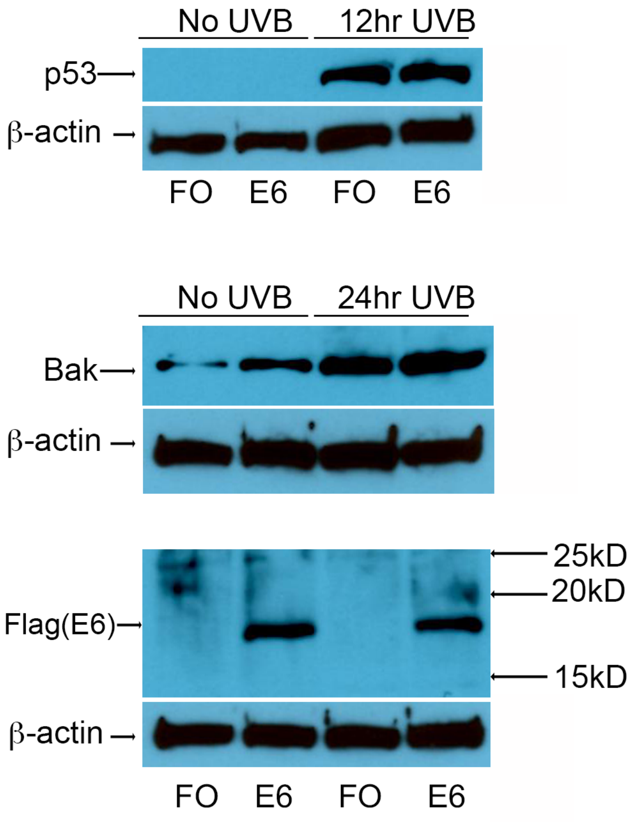

Supplemental Figure 1. Canine primary epidermal keratinocytes expressing CPV2E6 with an N-terminal 3xFLAG epitope (E6) or Flag only (FO) were exposed to UVB (10mJ/cm^2) and cell lysates collected at 12 and 24 hours post-irradiation. Proteins were separated by SDS-PAGE and analyzed by immunoblotting with antibodies directed against p53, Bak, and Flag. β-actin was used a loading control. One representative experiment of two is shown.

Supplemental Methods:

To construct the 3xFLAG-CPV2E6 plasmids, PCR (HF Clonteq taq) was performed using extracted DNA from a clinical papilloma sample that contains wild-type CPV2 E6 using the following primers: For 5' GCG CTC GAG GCC GCC ACC ATG GAC TAC AAA GAC CAT GAC GGT GAT TAT AAA GAT CAT GAC ATC GAT TAC AAG GAT GAC GAT GAC AAG GGT GGA GGC GGT AGC GCG CGG CCA CTG AGC ATA and Rev 5' CTC GAA TTC TTA CTA CAC GCA GCT GTC ACA AGT GTT CC. The forward primer contains a restriction site for *XhoI*, kozak sequence, three FLAG tags, and linker sequence. The reverse primer contains a restriction site for *EcoRI*. The resulting amplicon was purified using a commercially available kit (Promega). The Flag only plasmid was generated by annealing the following two oligos which contain cohesive XhoI and EcoRI sites and three FLAG tags: Top oligo 5' TCG AGG CCG CCA CCA TGG ACT ACA AAG ACC ATG ACG GTG ATT ATA AAG ATC ATG ACA TCG ATT ACA AGG ATG ACG ATG ACA AGT AAA CTC G and Bottom oligo 5' AAT TCG AGT TTA CTT GTC ATC GTC ATC CTT GTA ATC GAT GTC ATG ATC TTT ATA ATC ACC GTC ATG GTC TTT GTA GTC CAT GGT GGC GGC C. Oligos were dissolved in ddH_2O to reach concentration of 1 μg/μl. A 20 μl annealing reaction was performed with 0.36 μl top-strand oligo; 0.36 μl bottom strand oligo; 1 μl 20X SCC (Sigma, Cat S6639), and 18.28 μl water. The mixture was heated to 95°C for 10 minutes, left at room temperature for 1 hour, and then diluted to a final insert concentration of 40 ng/μl with 30 μl of TE buffer. Restriction enzyme digestion with XhoI and EcoRI was performed on the retrovirus vector backbone MSCV and the purified PCR product, followed by purification by agarose gel electrophoresis. MSCV was a gift from Lin He (Addgene plasmid # 24828 ; http://n2t.net/addgene:24828; RRID:Addgene_24828).[1]

The vector backbone was routinely ligated to the coding sequence of 3xFLAG-CPV2E6 or the 3xFLAG only annealed oligos. Retrovirus stocks were prepared by transfecting the retrovirus packaging cell line PT67 (kind gift Cary Moody) with flag only or 3XFlagCPV2E6 retrovirus constructs using TransIT X2 (Mirius) as specified by the manufacturer. All plasmids were sequenced to verify the correct nucleotide sequence of the constructs. PT67s were selected for 10 days in 2 μg/ml puromycin. After selection, PT67s were seeded into 10cm^2 culture dishes at 1:20 and 1:40 in 10 mls DMEM containing 10% FBS. After three days, the supernatants were collected and retrovirus stocks concentrated using the Retrovirus concentrator following their recommended protocols. CPEKs were transduced in 10cm^2 dishes with 2 mls concentrated retrovirus, 5 μg/ml polybrene (Santa Cruz SC-134220), and 3 mls CNT-09 keratinocyte culture medium. Two days post-transduction, cells were selected for 10 days in 1 μg/ml puromycin.

Ultraviolet exposure was performed as described previously in the methods, followed by protein isolation. Immunoblots for p53, Bak, and β-actin were performed as previously described in the methods. Immunoblots were probed with mouse monoclonal antibody against FLAG (Cat #: F-1804, Sigma) at 1:400 dilution overnight at 4°C followed by application of horseradish peroxidase-conjugated mouse (Pierce Biotechnology Inc., Thermo Fisher Scientific) secondary antibody at 1:5000 for 1 hour. Detection was made using a chemiluminescence reagent (GE Healthcare, Chicago, IL), followed by exposure of the membrane to film.

1. **miR-19 is a key oncogenic component of mir-17-92**. Olive V, Bennett MJ, Walker JC, Ma C, Jiang I, Cordon-Cardo C, Li QJ, Lowe SW, Hannon GJ, He L. *Genes Dev. 2009 Dec 15. 23(24):2839-49.* 10.1101/gad.1861409 PubMed 20008935
