# Supplementary figures and images for: Canine Papillomavirus 2 E6 Does Not Interfere With UVB-Induced Upregulation of p53 and p53-Regulated Genes
Source: Front Vet Sci. 2021 Mar 3;8:570982. doi: 10.3389/fvets.2021.570982 (PMC7965962; doi:10.3389/fvets.2021.570982)

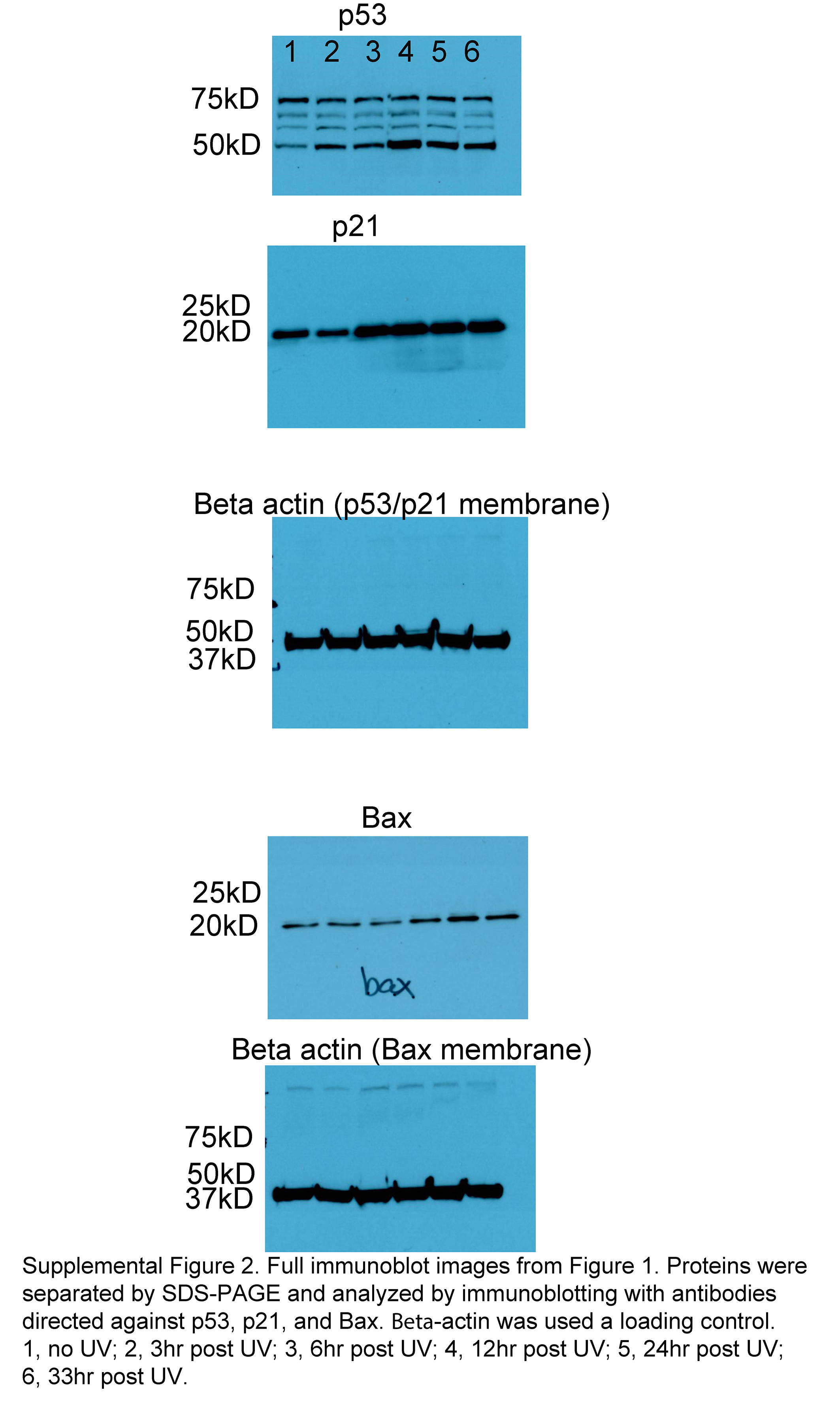

Supplement: Supplementary file 2 [file Image_1.tif]

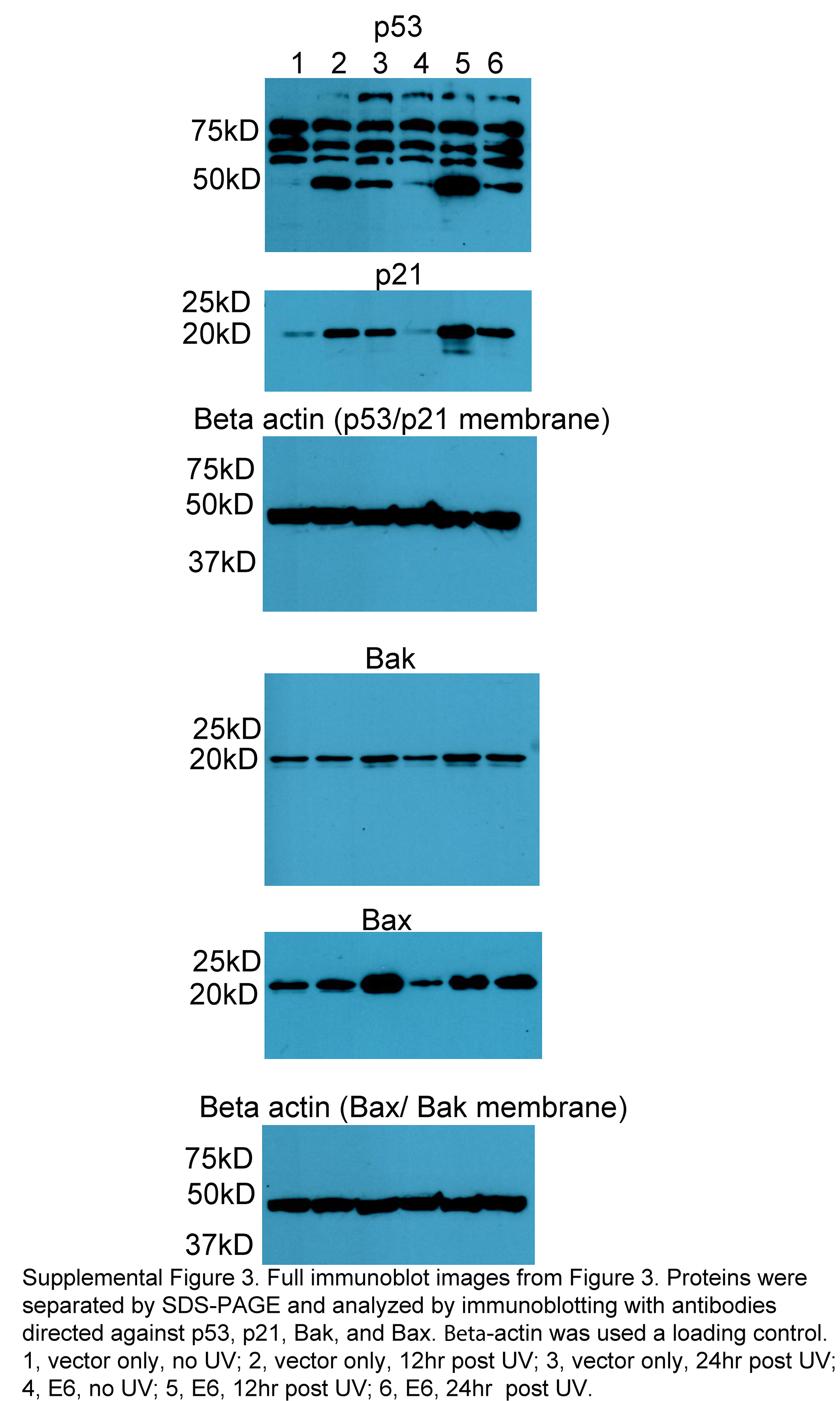

Supplement: Supplementary file 3 [file Image_2.tif]
